# ‘Yellow is good for you’: Consumer perception and acceptability of fortified and biofortified cassava products

**DOI:** 10.1371/journal.pone.0203421

**Published:** 2018-09-14

**Authors:** Aurélie Bechoff, Ugo Chijioke, Andrew Westby, Keith Ian Tomlins

**Affiliations:** 1 Natural Resources Institute (NRI), University of Greenwich, Chatham, Kent, United Kingdom; 2 National Root Crop Research Institute, Umudike, Umuahia, Abia State, Nigeria; Shanghai Institutes for Biological Sciences, CHINA

## Abstract

Vitamin A, an essential micronutrient for health, can be obtained from various food sources including cassava products made from either traditional white cassava varieties fortified with red palm oil containing provitamin A, or new high provitamin A biofortified yellow cassava varieties. Both products have a similar yellow appearance due to the coloured pigmentation of provitamin A. Using a range of methods to provide a comprehensive understanding of sensory acceptability (blind triangle test, sensory profiling, hedonic preference that included Check-all-that-applies and Just-about-right tests), we tested the acceptability and nutritional perception of traditional West-African food dough-like products (eba and fufu) made from biofortified, fortified, or control products made with non-fortified white cassava (n = 7) at three suburban locations near Ibadan, Nigeria on a total of 122 consumers. Biofortified, fortified, and control products could be differentiated blindly confirming that products clearly differed with respect to other sensory characteristics than appearance. Overall biofortified products were better accepted than control and fortified ones. Three classes of consumer preference were identified based on the dislike for control and fortified products, which indicated that acceptance of biofortified products was not a hindrance. On the contrary the traditional fortified product had poorer acceptance and this was due to its less desirable sensory characteristics as demonstrated by Just-about-right Penalty analysis. A majority of consumers (85%) had previous knowledge of biofortified cassava. Consumers associated ‘yellow colour’ with ‘good for eyesight’, ‘good for children’s health’ and ‘new’. More nutritional benefits were attributed to biofortified than fortified products although they had similar provitamin A contents and this demonstrates a bias. We suggest that nutrition promotion campaigns to improve the vitamin A status should also encompass all natural sources of provitamin A, including biofortified and traditional fortified products.

## Introduction

Cassava (*Manihot esculenta* Crantz), a drought resistant root, is a subsistence crop for millions of people in sub-Saharan Africa. The daily consumption of cassava can reach up to 940 grams per adult per day [1]. A constraint of cassava however is that its overall nutritive value is poor: the root is mainly composed of starch that is a good source of energy, but contains few other nutritive elements such as proteins or micronutrients [2]. Together with zinc and iron deficiencies, vitamin A deficiency (VAD) is one of the main world micronutrient deficiencies and is linked to high child mortality in countries where the diet is not rich enough in vitamin A. In Nigeria, the world’s largest producer of cassava and the most densely populated country in Africa, the prevalence of VAD among children 0–59 months of age is 30% [3].

New varieties of cassava that are rich in provitamin A carotenoids (pVACs) have been developed [4]. These biofortified cassava varieties have yellow flesh due to their carotenoid content and pVACs are more bioavailable than in other crops [5–8]. Therefore there is strong expectation that these varieties could help tackle VAD in sub-Saharan Africa and other parts of the world where cassava is consumed.

The nutritional value of cassava products can also be improved by the use of red palm oil (RPO). Addition of RPO is a traditional practice in some parts of Nigeria (e.g the south-east) that helps confer pVACs [9] to the products made from white cassava. The colour and the pVAC contents of products from white cassava with RPO are similar to that of biofortified cassava [9]. Furthermore, it has been demonstrated that the bioavailability of pVACs was greater in products fortified with RPO compared to those made with biofortified cassava [7].

Consumer acceptability of biofortified cassava has been described in a number of studies: for instance, willingness to pay for genetically modified (GM) biofortified yellow cassava in Brazil was examined [10]. It was revealed that the consumer acceptability of the products was affected by the knowledge that the cassava was GM and the authors suggested that the acceptance of conventionally bred cassava would be higher. Talsma et al. [11] investigated the acceptability of conventionally bred biofortified cassava with schoolchildren in Kenya and their caretakers. In this study, 97/1170 variety (yellow colour) was tested against a local cassava variety of white colour. The product was simply boiled and pureed; the method of preparation indicated that the variety must have been a sweet type (containing low levels of cyanogenic compounds) that is popular in East-Africa. The acceptability was assessed using a replicated discrimination test and a paired preference test. In addition cultural acceptability of the product was tested using a Likert scale to assess the perception of different perception statements about yellow cassava. A difference in taste between yellow biofortified and white traditional cultivars was perceived by a blind test. Results showed that puree from biofortified cassava was well received and was overall more acceptable than that from local cassava by schoolchildren.

Many varieties of cassava are toxic because they contain significant amounts of cyanogenic compounds (bitter varieties), and therefore require extensive processes to make them safe for human consumption [12]. Because of the many processing steps involved, processing cassava into gari and fufu products are ways of making bitter cassava safe for consumption. Gari is a semolina-like dried product with a slight acidic taste that can be rehydrated by addition of boiling water and made into a dough called eba. Fufu is a wet form of cassava that is fermented, sieved to remove fibres, and made into a cooked dough. Gari and fufu are the main sources of carbohydrate in the Nigerian diet, with gari representing two thirds of the fresh cassava production [13]. An study by Oparinde et al. [14] explored the acceptability of gari and eba products from bitter biofortified varieties of cassava (TMS 01/1368 and TMS 01/1371) in two different states in Nigeria. Results showed a positive incentive toward biofortified cassava products as compared with traditional products. However the focus of the research was on the willingness to pay [15], and in-depth research is still needed to understand what the relationships between sensory characteristics and consumer acceptability are in order to predict market needs.

In our study, we investigated the sensory characteristics and consumer acceptability of biofortified cassava products versus commonly consumed white cassava and RPO-fortified white cassava. We used conventionally bred yellow biofortified and bitter cassava varieties made into major traditional food products, eba (made from gari) and fufu. Our purpose was to understand: (1) how consumer acceptability is affected by products having the same colour (from biofortified cassava and white cassava fortified with RPO) or a different colour (from biofortified cassava and white cassava) and (2) whether consumers are aware of the link between yellow colour and nutritional benefits due to the pVAC content of the products.

## Materials and methods

### Ethics

This study was assessed and approved by the University of Greenwich Research Ethics Committee. Samples were prepared according to good hygiene and manufacturing practices. Participants were informed about the study and explained that their participation was entirely voluntary, that they could stop the interview at any point and that the responses would be anonymous. Written consent (signature) was sought from sensory panellists and from consumers participating in this study.

### Cassava root supply

This research work was a follow-on of a study on the retention of carotenoids in biofortified cassava varieties during gari processing that was carried out the year before [16]. ‘First wave’ biofortified varieties of yellow-fleshed cassava released by HarvestPlus-Nigeria—TMS 01/1368 (yellow-flesh colour) (8–10 μg.g^-1^ total carotenoids including 3–4μg.g^-1^ trans-β-carotene with an average total cyanogenic potential (CNp) between 75–148 ppm) [17]; and TMS 01/1371 (deep yellow-flesh colour) (7–8 μg.g^-1^ total carotenoids including 5–6 μg.g^-1^ trans-β-carotene with an average total CNp between 85–132 ppm) [17] and local variety (TME 419) (white) (values were not measured. According to local sources, TME 419 roots contain approximately 1 μg.g^-1^ total carotenoids including 0.3μg.g^-1^ trans-β-carotene and have a CNp around 40–60 ppm) were used. The three varieties were grown at Ikenne (6°86N, 3°71E) in South-West Nigeria TME 419 was planted on the 16^th^ July 2012 and TMS 01/1368 and 01/1371 on 9^th^ August 2012. All three varieties were harvested on the 18^th^ November 2013. No specific permissions were required because HarvestPlus/IITA had the authorisation to use those lands for research purposes. The study did not involve endangered or protected species.

### Processing of roots

All roots (50kg per variety per product (for fufu or gari)) were brought by road and processed on the day following the harvest on the processing platform at the International Institute for Tropical Agriculture (IITA), Ibadan, Nigeria.

Because the yellow varieties were bitter (CNp > 50ppm), a simple boiling would not have been sufficient to remove cyanogens to a safe level (<10 ppm) and extensive processes such as fufu and gari making were required. During processing of the different varieties, there was a step-by-step monitoring of initial and final quantities, ambient temperature/humidity, length of time, pH values (after fermentation) (Table 1).

**Table 1:** Processing conditions for fufu and gari products from biofortified (yellow) and local (white) varieties.

Product		Fufu			Gari (for Eba)			
Variety		TMS 01/1368	TMS 01/1371	TME 419	TMS 01/1368	TMS 01/1371	TME 419	TME 419 + RPO
**Abbreviation**		**BP-F1**	**BP-F2**	**C-F**	**BP-G1**	**BP-G2**	**C-G**	**FP**
**Biofortified**		Yes	Yes	No	Yes	Yes	No	No
**Fortified**		No	No	No	No	No	No	Yes
**Weight**	**Initial**^**a**^	50.4	50.1	50.4	50.5	50.1	50.6	50.2
	**Final**^**b**^	13.3	11.8	21.3	4.4	5.4	8.7	9.3
**T(°C)**		25.6	25.8	25.1	25.8	25.1	26.1	25
**pH**		4.8	4.3	5.1	4.0	4.0	4.0	4.0
**Processing time (h)**	**Peeling**	0.92	1.33	1.13	0.82	1.55	1.3	0.83
	**Washing**	0.30	0.22	0.22	0.15	0.28	0.23	0.3
	**Size reducing**	0.13	0.15	0.13	-	-	-	-
	**Piece soaking**	44.65	44.65	44.65	-	-	-	-
	**Washing**	0.10	0.10	0.10	-	-	-	-
	**Grating**	0.03	0.07	0.07	0.05	0.08	0.08	0.07
	**Mash fermenting**^**c**^	22.88	23.05	23.12	44.30	44.18	44.08	44.52
	**Sieving**	0.75	0.82	0.73	-	-	-	-
	**Pressing**^**d**^	3.92	3.92	3.92	5.03	5.03	5.03	5.03
	**Sifting**	-	-	-	0.03	0.02	0.07	0.02
	**Roasting**	-	-	-	0.73	0.77	1.03	0.70
	**Sieving**	-	-	-	0.08	0.10	0.10	0.10

Each process was conducted in one batch. 50kg of roots were used to process fufu and gari. Process was conducted at the IITA processing plant.

^a^Unpeeled roots

^b^pressed mash for fufu or sieved gari

^c^mash fermenting for gari or fufu

^d^using manual press for fufu & hydraulic press for gari

Roots were peeled on the same morning for the processing of gari and fufu:

The processing steps for gari were: peeling, grating, mash fermenting, pressing and roasting. Fermentation was two days (about 44h). The local variety (TME 419) was processed with and without red palm oil (RPO). RPO was bought on the market in Ibadan.

The processing steps for fufu processing were: peeling, piece soaking (fermenting of peeled pieces of cassava), grating, mash fermenting, sieving, and pressing. Fufu (as pressed mash) and gari (to be made into eba) were stored at -20°C and used for sensory and consumer tests in the following two weeks. In our experiment, freezing was used for logistic purposes however it has been reported for products made at commercial scale.

### Preparation of products for sensory evaluation and consumer analysis

Frozen pressed mash for fufu was collected from the -20°C room and left to thaw overnight. The mash was cooked the following morning into fufu by adding water and cooking on the gas cooker at the IITA cooking laboratory.

Gari was collected on the morning from the freezer, thawed at ambient, and made into eba by adding boiling water, and the mixture (gari + hot water) was then pounded using a traditional African mortar and pillar to make it into a homogeneous paste (eba).

### Products

The products (n = 7) were the following:

Eba from gari (n = 4):

TMS 01/1368 (biofortified bitter variety of yellow colour) (BP-G1)

TMS 01/1371 (biofortified bitter variety of yellow colour) (BP-G2)

TME 419 (local variety of white colour) (C-G)

TME 419 with red palm oil (RPO). Product was yellow in colour because RPO contains carotenoids (FP)

Fufu (n = 3):

TMS 01/1368 (BP-F1)

TMS 01/1371 (BP-F2)

TME 419 (C-F)

Gari can be consumed either as dried (gari) or as wet dough (eba). Eba is the most common form of consumption of gari therefore the study focused on eba. Whilst eba can be prepared with RPO that confers yellow colour to the product, fufu is not usually prepared with RPO. Yellow fufu (from biofortified cassava) is therefore a novel product since the traditional form of fufu is not normally yellow.

Samples were served to consumers after cooling down at ambient temperature. Maintaining samples warm would have required tight logistics procedure and eating fufu or eba at ambient temperature is a usual practice in Nigeria. The samples were served everyday around the same time therefore if the texture had been affected by cooling, the effect would be the same on each day.

### Blind triangle test

Students and staff volunteers from IITA (n = 12) were individually blindfolded and presented with three samples, with two of which being identical. Two varieties, TMS 01/1371 (yellow biofortified (BP)) and TME 419 (white andlocal- used as control (C)) were tested with three different combinations:

1. A = Eba TME419 (C-G); B = Eba 01/1371 (BP-G2).
2. C = Fufu TME 419 (C-F); D = Fufu 01/1371 (BP-F2)
3. E = Eba TME419 + RPO (FP); F = Eba 01/1371 (BP-G2).

Subjects were asked if they detected that samples were different. If they did, they were then asked to designate which sample was the odd one. Samples were presented in a random order to each subject. This experiment’s aim was to determine if consumers might be able to differentiate yellow and white cassava products without seeing the colour (It should be noted however that cassava of the same colour may also have different texture, smell, and taste).

### Sensory evaluation

Products (eba, fufu) were scored by a semi-trained sensory panel using a modified version of quantitative descriptive analysis (QDA) since standards were not provided [18, 19]. The panel was composed of university students from various States of Nigeria (n = 11) on placement in IITA. Sessions were conducted at the IITA sensory laboratory. The language used for the sensory testing was English. The panellists were all familiar with processed products from cassava roots. Focus groups were carried out prior to the evaluation for eba and fufu. Sensory attributes were generated during these preliminary focus group session guided by the panel leader. Sensory attributes (11) were developed in consensus with panellists. Sensory attributes generated were as follows:

- Yellow colour: dough that is yellow in colour
- White colour: dough that is white in colour
- Fresh gari smell: dough that has the pleasant smell of freshly made gari
- Fermented fufu odour: dough that has the odour of fermented fufu product
- Smooth texture: dough that is homogeneous in appearance and hand feel and does not have fibres, lumps or particles
- Soft texture: dough that is soft in texture
- Elastic texture: dough that has a texture that tends to come back into place after stretching it
- Mouldable texture: dough that can be made moulded like clay
- Sticky texture: dough that is adhesive or gummy to the hand
- Fresh gari taste: tastes of fresh gari neither sour nor bland
- Sour taste: tastes of lime–expressed as ‘slaps your cheek’

After a period of training using these attributes, the samples from the three different products were tested in triplicate by the panel and the order in which they were presented was random. At each session, four samples coded with 3-figure random numbers were served in a different random order to each panellist so that the panellists did not know which sample they were testing. Eba and fufu products (~20g) were presented in cling film bags and served at ambient temperature. The panellists were asked to open the bags or containers and score the intensity for the sensory attributes on a 108 mm unstructured scale, anchored with the terms ‘not very’ at the low end and ‘very’ at the high end.

### Consumer acceptability

Consumer acceptability tests were conducted in suburban areas of Ibadan using the same products as for sensory evaluation. Local people who consume cassava as a staple food were asked their opinion about the products. A minimum number of 60 consumers are required for a consumer acceptability study but the preferable number of consumers is 100 [20]. Since more tests including sensory and emotional perceptions of consumers—such as Check-All-That-Apply (CATA) and Just-About-Right (JAR)—were added to the questionnaire, additional consumers were beneficial to the quality of the study and we targeted a number of n~120 consumers. Only adult consumers (over the age of 18) were selected.

**Fig 1:**
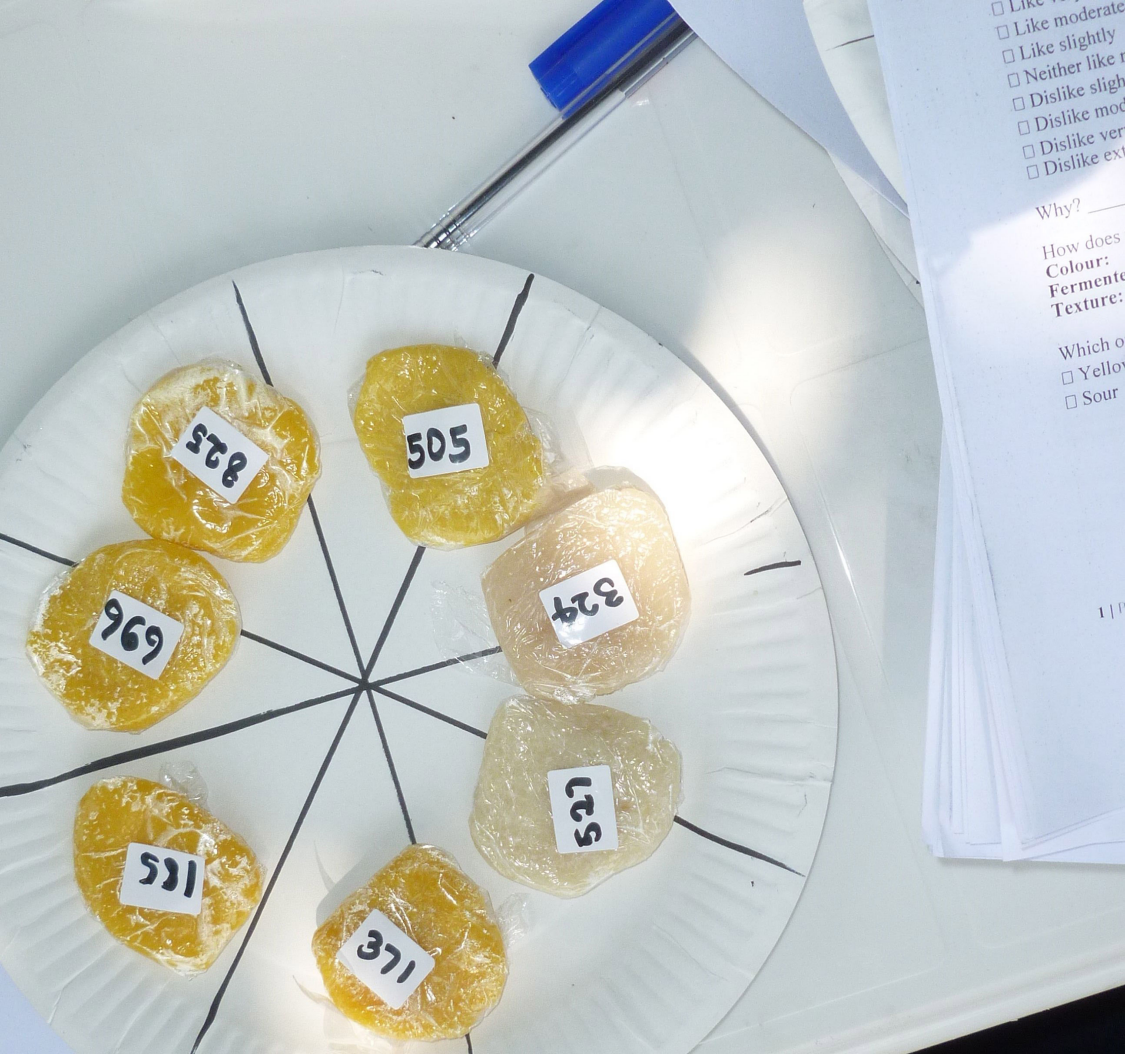
Fufu and eba samples ready for consumer acceptability tests. 969: Eba TMS 01/1368 (yellow) (BP-G1); 371: Eba TMS 01/1371 (yellow) (BP-G2); 527: Control Eba TME 419 (white) (C-G); 505: Eba TME 419 with RPO (yellow) (FP); 825: Fufu TMS 01/1368 (yellow) (BP-F1); 185: Fufu TMS 01/1371 (yellow) (BP-F2); 324: Control TME 419 Fufu (white) (C-F). Note: the cling film was removed before the consumers assessed the products.

A total of consumers (n = 122) were randomly interviewed at three different locations using the central location method [18]. These sites were target villages where HarvestPlus had recently distributed cassava cuttings and the aim was to look at the perception of yellow cassava by areas where promotion of biofortified cassava had taken place. The number of consumers per location was: Ojutaye (n = 39); Omi Adio (n = 40); Lalupon (n = 44). Samples were cooked, packed and labelled in the morning and carried in a cool box to the consumers. All the samples (n = 7) were presented to the consumers on a single plate (Fig 1).

During acceptability testing, each consumer was invited to taste each fufu or eba sample (~20g presented in random order and coded with three figure random numbers). Consumers opened samples wrapped in film. Consumers were then asked to score the overall liking (with respect to appearance and taste) using a nine-point verbal hedonic box scale which varied from dislike extremely (1) to like extremely (9) [18].

Along with obtaining information about the acceptability of the fufu or eba products, information was elicited from each consumer regarding demographics, education, gari and fufu consumption.

Correlations between sensory profile and consumer acceptability and Check-all-that-applies (CATA) and Just-about-right (JAR) recent sensory techniques with consumers [21] were also applied in order to relate consumer acceptability to sensory characteristics and understand the drivers of acceptance of these traditional products from biofortified cassava. A Check-all-that-applies (CATA) and a Just-about-right (JAR) (3 levels) tests help describe the samples’ attributes and feelings associated to the eating of the products. CATA is a technique to help understand the likings of consumers that was developed by Lancaster & Foley [22] and became more popular for use in consumer acceptability under Ares et al. [23]. A CATA questionnaire consists of a list of words or phrases from which consumers should select all the words to describe a product. Just about right (JAR) scale combines hedonic and sensory perception. In some cases JAR scale is useful to help understand the reasons behind hedonic scoring of products and relate hedonic scoring to specific sensory characteristics. These scales are bipolar having two opposite end-anchors (for example: too soft / too hard). The centre point is JAR. The consumer will then have to choose between three choices (e.g. ‘too soft’; JAR; or ‘too hard’).

JAR and CATA sensory attributes were generated during focus group sessions guided by the researcher. Attributes for JAR (6) and CATA (8) were developed in consensus with our local collaborators.

JAR attributes were:

- Colour: too white; JAR; too yellow
- Fermented odour: too weak; JAR; too strong
- Texture: too soft; JAR; too hard.

As well as JAR, CATA also combines a sensory description of the products with consumer acceptability. But in addition to JAR technique or to QDA (sensory profiling), CATA can include questions about psychological and nutritional perception of products.

CATA attributes were as follows:

(For sensory perception)

- Yellow in colour
- Sour
- Good taste
- Mouldable
- Smooth

(For perception of novelty)

- New

(For perception of nutritional benefits on health)

- Good for children’s health
- Good for eye sight

All spoken interviews were conducted in English or in the local language (Yoruba) and the score sheets and questionnaires were written in English. Trained enumerators assisted the consumers when required. The interview procedure lasted no more than 30 min.

### Statistical analysis

Analysis of variance (mixed effect model), correlation analysis (Pearson), Chi-squared analysis and principal component analysis (correlation matrix) were carried out using SPSS (V 23.0) or XLSTAT (V 5.2, Addinsoft). Segmentation was done to explore the variation in consumer acceptability that exists within samples: hierarchical cluster analysis (Wards method), a method that divides consumers into groups of similar acceptance, was used to segment the consumers interviewed at the different locations into three different groups. Segmentation gives a more complex variation in acceptability among the consumers and is helpful to understand differences in consumer behaviour. Multiple pairwise comparisons were undertaken using the Tukey test with a confidence interval of 95%. Critical values at p < 0.05 for the triangle test were determined using a Triangle test statistical table.

The frequency of intensity ratings (CATA, JAR) was determined for each product for each of the sensory attributes evaluated by the consumers. A Penalty analysis [23] was used to relate CATA and JAR attribute intensity ratings to hedonic liking ratings. Significance of weighted penalties was evaluated by taking into account the weight mean drops and the proportion of participants (minimum 20% to be accounted for) in each group. A Friedman and a Cochran’s Q tests for JAR and CATA, respectively, were performed, for each descriptor to assess whether products significantly differed in terms of sensory attributes judged by the consumers (p<0.05).

## Results and discussion

### Discrimination test: Blindfolded triangle test

Initially a blindfolded triangle test was carried out to determine whether people could differentiate products from white or yellow cassava without the help of visual (colour) observation. The aim was to collect additional information on product differences that could not be captured directly by the consumer acceptability study. A majority of subjects (9/12) were able to differentiate the white (TME 419) (control) and biofortified (01/1371) eba products (p = 0.01) and 8/12 subjects could rightly differentiate the control and biofortified fufu (01/1371) with a significance of p = 0.05. Equally 8/12 subjects could differentiate between the fortified eba (FP) and the eba from 01/1371 biofortified cassava (BP-G2) with a significance of p = 0.05. Hence in all the combinations, subjects were able to correctly identify the odd sample. This shows that the products could be differentiated based on other sensory characteristics (taste, texture and smell) than the appearance: the products from biofortified cassava exuded distinct sensory characteristics to the white (local) one that were not only due to the different colour. In addition, the sample containing RPO could be perceived different from that of a biofortified variety of the same colour. The results showed that visual appearance was not the only difference between products of white and yellow cassava.

These results were in accordance with Talsma et al. [11]: in a blind test carried out with biofortified or local cassava puree, it was shown that 130/180 responses were correct and this means that subjects were able to discriminate between biofortified and local not based on the appearance. Using a blind triangular test with Quality Protein Maize (biofortified maize with high quality protein i.e. lysin and tryptophan), De Groote et al. [24] also showed that 70% of respondents were able to blindly differentiate QPM from local maize. In accordance with these results, Tomlins et al. [25] reported that the products from biofortified orange fleshed sweet potato of various colour not only vary in their colour but also in other sensory characteristics such as texture, taste, and odour. Products from yellow cassava were sweeter and softer than those from white cassava overall [26] Biofortified (BP) and non-biofortified products (C) can differ in other sensory characteristics than appearance (i.e. colour) only but this can also be the case for products from different varieties of sweet potato or cassava that have similar colour. In addition, the fortified product (FP) of similar colour to BP had distinctive sensory characteristics compared to BP, and C. Showing that those products can be distinguished on other criteria (*i*.*e*. taste and smell) that simple appearance (white or yellow) is important to understand consumer acceptability.

### Sensory profile

We conducted sensory profiling of the food products made with biofortified (BP) or local cassava (FP and C). The characteristics of fufu and eba products (n = 7) scored by the panellists are presented in Table 2.

**Table 2:** Means and probabilities for sensory testing with respect to eba and fufu sample and sensory panellist.

Attribute /Product	Eba 01/1368	Eba TME 419 (palm oil)	Eba TME 419	Eba 01 /1371	Fufu 01 /1368	Fufu 01 /1371	Fufu TME 419	Sample (S)	Panel-list (P)	S x P
*Abbreviation*	*BP-G1*	*FP*	*C-G*	*BP-G2*	*BP-F1*	*BP-F2*	*C-F*			
Yellow colour	83.8± 24.7bc	72.6± 18.4b	4.5± 18.7a	98.3± 14.9cd	98.1± 11.4d	91.8±19.2d	4.6± 18.7a	<0.001^a^	0.017 ^a^	0.477
White colour	11.6± 22.0b	11.5± 15.4b	89.7± 20.5c	2.5± 5.4a	3.5± 5.6ab	4.8± 6.4ab	85.6± 14.0c	<0.001^a^	0.245	<0.001^a^
Fresh gari smell	79.4± 33.7c	42.3± 40.4b	64.7± 35.2c	72.3± 24.9c	17.8± 26.7a	15.9±22.7a	13.0±23.7a	<0.001^a^	<0.001^a^	0.006 ^a^
Fermented fufu odour	12.2± 20.1a	18.8± 22.2a	18.0± 26.2a	13.6± 24.4a	71.5± 36.1b	71.6±35.1b	83.8± 32.3b	<0.001^a^	<0.001^a^	<0.001 ^a^
Smooth (appearance & hand)	42.5± 32.1a	55.5± 35.6a	48.9± 31.1a	45.6± 25.0a	98.6± 12.3b	91.0±18.9b	90.2± 22.5b	<0.001^a^	0.003 ^a^	0.010 ^a^
Soft texture (hand)	45.6± 30.6a	68.3± 31.3bc	44.2± 28.9a	52.2± 26.8ab	86.1± 29.2cd	91.4±19.9d	68.9± 33.7bc	<0.001^a^	0.039 ^a^	0.003 ^a^
Elastic texture (hand)	42.9± 33.2bc	11.2± 19.2a	75.4± 34.1d	25.8± 26.5ab	54.2± 38.2c	44.5±35.2c	98.6± 11.6e	<0.001^a^	<0.001^a^	0.012 ^a^
Mouldable texture (hand)	72.6± 25.8bc	38.9± 35.5a	83.6± 25.9c	60.2± 31.0b	78.2± 28.4c	75.8±29.3bc	86.0±28.3c	<0.001^a^	0.001 ^a^	<0.001^a^
Sticky texture (hand)	48.5± 35.7abc	29.6± 36.9a	65.9± 37.1cd	43.5± 29.8ab	67.2± 33.3cd	78.2±30.6d	60.3± 44.6bcd	<0.001^a^	0.001^a^	<0.001^a^
Fresh gari taste	71.2± 29.5b	22.6± 32.9a	63.6± 33.9b	60.4± 32.1b	25.1± 32.7a	19.1±25.2a	18.8± 28.1a	<0.001^a^	0.013 ^a^	0.001^a^
Sour taste	48.7± 33.5ab	42.0± 32.8a	59.8± 34.1ab	67.9± 33.0bc	57.2± 36.8ab	85.5±26.4c	68.3± 32.0bc	<0.001^a^	<0.001^a^	0.015^a^

^a^ indicate significant differences. Intensity of sensory attributes was scored a 100mm scale. Average ± standard deviation. Differences between the samples (a, b, c, d or e in columns) were determined by the Tukey method

The sensory attributes of the seven eba and fufu products tested were strongly significantly different with respect to sample (linear mixed model; ANOVA; p<0.05) for all the sensory attributes (yellow and white colours, fresh gari smell and taste, fermented fufu odour, smooth appearance, soft, elastic, mouldable, sticky textures, sour taste) (column S). Hence it means that the panellists were able to differentiate all the products’ attributes.

Significant sample panellist interactions (column S x P) for some of the attributes (p<0.05) are probably because it was not possible to provide standards and because the panel was semi-trained and some attributes were more difficult to assess; such observations are common even with trained panellists [27].

Yellow and white colours were unmistakably attributed to biofortified and local products respectively. The yellowest samples were BP-F1 and BP-F2 fufu products followed by BP-G2 eba. RPO-fortified eba (FP) was also yellow and perceived having colour intensity similar to that of BP-G1. ‘White colour’ was reverse to ‘Yellow colour’ in accordance to what was expected. To our knowledge little work has been reported on the description of sensory attributes from biofortified cassava using Quantitative Descriptive Analysis (QDA). However other methods have been used: a participatory approach was used for farmers to describe the desirable characteristics of biofortified cassava [28] and the selection of sensory descriptors was done by consumers in another study [11]. Tomlins et al. [29] used the QDA method to describe the sensory profile of non-biofortified cassava fufu that were either fresh or dried and some of the descriptors were similar: ‘soft’, ‘sour’, ‘smooth appearance’, ‘sticky’ and those were related to texture however there was no description of the variation in colour (*i*.*e*. white to yellow) since the cultivars used were all white or cream. On the other hand when working with biofortified orange-fleshed sweet potato, a range of colour (‘cream’, ‘yellow’, ‘orange’) were reported as sensory descriptors [25].

Fresh gari smell and taste, and fermented fufu odour were clearly attributed to eba, and fufu, respectively, and this shows that the panellists were able to differentiate the two products based on their smell. When RPO was present in the eba, the perception of the fresh gari smell was not as pronounced and this must be because it was masked by the RPO smell. Fermented fufu odour was clearly more intense in fufu products than in gari products. Fermented fufu odour was strongly associated with fufu from 01/1368 and 01/1371 varieties (BP-F1 and BP-F2, respectively). Since all the fufu samples were fermented the same length of time and under the same conditions, the stronger odour of fufu (fermented smell) in the biofortified varieties could be explained by their lower starch level and therefore their higher ability to ferment compared to the traditional white variety (TME 419). Fresh gari smell and taste were associated with eba from 01/1368 and 01/1371 varieties (BP-G1 and BP-G2, respectively) but also eba from the white variety (C-G).

Fufu products were also smoother and with softer texture than the eba products and this may be attributed to the fact that fibres had been removed from the fufu products whilst they were still present in the eba products. The most elastic products were the white eba and fufu and this seems to indicate a higher quality of starch or higher dry matter content. The white products were also the most mouldable although there was no significant difference in mouldability with the other products. The stickiest were the white eba and the three fufu products. Variety TMS 01/1371 produced the sourest samples (eba and fufu).

Samples of gari, fufu and eba from different cassava root colours (white or yellow), with or without palm oil differed in most of their sensory attributes. The profile obtained using QDA was distinct for the samples of gari and eba, fufu and this will help when it comes to understand the consumer acceptability of these samples.

### Consumer acceptability

#### Socio-demographic characteristics of the consumers

The main socio-demographic characteristics and cassava food habits of consumers were reported (Table 3).

**Table 3:** Demographic differences and consumer attitudes to eba and fufu.

Question		All consumers
Number of inteviewees		122
Location (3 villages)	Lalupon	36.1%
	Ojutaye	32.8%
	Omi Adio	32.8%
Male		49.1%
Yoruba tribe		96.7%
Age (years)	Average (min-max)	43.9 (18–83)
Education	No education	25.4%
	Primary	33.6%
	Secondary	38.5%
	University	3.3%
Occupation	Farmer	41.0%
	Trader	24.6%
	Skilled (hairdresser, barber, fashion designer…)	18.9%
	Professional (doctor, vet…)	3.3%
	Unskilled	8.2%
	Cassava processor	1.6%
	Student	1.6%
Staple	Cassava	81.1%
	Yam	7.4%
	Rice	8.2%
Main food crop	Gari	32.0%
	Lafun	54.1%
	Fufu	12.3%
Frequency of consumption per month	Gari	23.3 days
	Fufu	17.3 days
Form of gari consumed (%)	Eba	55.7%
	With milk or water	33.9%
	Dry	9.6%
Main colour of gari consumed	White	91.0%
	Yellow	4.1%
	Both	4.9%
Awareness of vitamin A (biofortified) cassava	Yes	86.9%
How did you hear about biofortified cassava?	Extension agent	45.1%
	Radio	12.3%
	Friend or neighbour	9.0%
	External organisation (i.e. HarvestPlus, IITA)	19.7%
Activity with biofortified cassava	Growing	24.6%
	Processing	15.6%
	Selling	1.6%

The number of people interviewed in each village was similar. The number of interviewees was also gender balanced since 49.1% were male. Almost all were from Yoruba origin (96.7%) the main tribe in this part of Nigeria (South-West). On average interviewees were middle-aged (43.9 years) and a large age variation was observed: 18 to 83 year-old. A majority were farmers (41.0%), followed by traders (24.6%), skilled workers (18.9%) and there were only a few cassava processors (1.6%). Cassava was their main staple (81.1%) followed by rice (8.2%) and yam (7.4%). Lafun (dried cassava flour reconstituted into a paste) was the main food (54.1%) followed by gari (32.0%) and fufu (12.3%). Gari was consumed most days (23.3 days in a month) and fufu every other day (17.3 in the month days). Most of the gari was prepared reconstituted into eba (55.7%), then some was prepared with addition of milk or water (33.9%), and also a small proportion was eaten as it is (9.6%). The gari consumed was by most of white colour (91.0%) however a small proportion consumed fortified yellow gari with RPO as their main form (4.1%) and others consumed both white and yellow gari with RPO (4.9%). This shows that gari of yellow colour was familiar to people. A majority of interviewees (86.9%) were aware of the biofortified cassava and 45.0% of them had heard about it through an extension agent, and 19.7% through HarvestPlus, IITA and other external organisations. Radio was cited by 12.3% and word of mouth (friends or neighbours) by 9.0%). Those who were involved with biofortified cassava actively were 24.6% growing it, 15.6% processing and a small proportion 1.6% selling it. High awareness about biofortified cassava and involvement in related activities can be explained because the three locations used in the survey were part of the targeted areas of HarvestPlus for the promotion of biofortified cassava.

#### Overall acceptability of products

Overall acceptability by the 122 consumers was determined on the different products (Table 4).

**Table 4:** Overall consumer acceptance of the different products.

Product	Acceptance^a^
Eba TME 419 (palm oil) (FP)	4.9±2.6d
Eba TME 419 (C-G)	6.4±2.1c
Fufu TME 419 (C-F)	6.8±1.9bc
Fufu 01/1371 (BP-F2)	7.0±1.8abc
Eba 01/1368 (BP-G1)	7.4±1.4ab
Fufu 01/1368 (BP-F1)	7.4±1.6ab
Eba 01/1371 (BP-G2)	7.7±1.2 a

^a^average±standard deviation (n = 122 consumers). Acceptance scale is between 1 and 9. Average was ranged from lowest to highest acceptability. Differences between the samples (a, b, c or d in columns) were determined by the Tukey method

Acceptance of cassava products from roots significantly differed between the seven samples at p<0.05 (One-way ANOVA). All of the products except the fortified eba (FP) were on average acceptable since the mean scores were much greater than a score of 5 (neither like nor dislike). The acceptance of FP was on the borderline with an acceptance of 4.9.

The low score for FP may also be because of its sensory properties but possibly because most of consumers consumed white (91%) gari and only 4% consumed yellow or 4% either yellow or white gari (Table 3).

The most liked was BP-G2 followed by BP-F1, BP-G1, BP- F2, C-F, C-G and lastly FP. The average liking for the samples was 6.8 (~score of 7 = ‘like moderately’), which is high. Tomlins et al. [29] working on the acceptance of fufu either from fresh dough (paste) or flour by Nigerian consumers reported liking values up to 6.9 for the fufu from fresh dough. The lowest acceptance score was 4.8 with fufu from flour. The scores reported in this present study are for fresh fufu (made from dough) (C-F = 6.8) and are in accordance with these findings [29]. Eba and fufu products from biofortified cassava were more acceptable on average (7 or more) than the equivalent products from white cassava. Earlier acceptance studies with other crops such as puree from biofortified sweet cassava varieties [11] showed a similar outcome. An interesting consumer acceptance study was conducted with different age groups for yellow biofortified maize in South Africa [30] reported that biofortified maize was more acceptable than white maize for preschool children but the trend was reversed when children were older and for adults. It has been conveyed that the yellow colour had negative connotation in the case of maize in East and Southern Africa because the consumption was associated with emergency food programs and therefore perceived as a poor man’s food. This explains why the acceptability was lower with older children and adults who are aware of these emergency programs. However in another study exploring the potential of orange biofortified maize in Mozambique, the orange colour was not a hindrance to acceptance [31] and this may have been because there was no negative perception associated with orange maize as opposed to yellow maize. In this present study with adult consumers acceptance of yellow cassava cultivars was higher to that of local white varieties and this was in accordance with an earlier study targeting school children [11]. This finding was also confirmed in a recent review on the acceptance of biofortified crops [32].

It is possible that prior knowledge of consumers about biofortified cassava could have boosted the acceptance scores. In a study on gari and eba from biofortified cassava in Oyo and Imo State, Oparinde et al. [14] reported that acceptability significantly increased when nutrition information was given to consumers. Nevertheless, another study by Oparinde et al. [33] on high-iron bean showed that the length of information (short or long) given to consumers did not influence the willingness to pay for the beans. It has even been described that too much information could also decrease the acceptability of biofortified crops [34]. On the other hand, repeated exposure to a food clearly results in increased acceptability [35, 36]. Therefore increased exposure and balanced amount of nutrition information may contribute to increase consumer acceptance and consumption of biofortified cassava products.

#### Segmentation of consumers into groups of similar acceptance pattern

Hierarchical cluster analysis (Wards Method) approach has been commonly used in consumer acceptance in order to determine which groups of people (and size) who would prefer which type of product. This approach is very useful when launching a new product on the market because it helps target specific consumers with the type of product they like. The liking can be depended upon many factors (socio-economic background; food customs) and knowing the consumer segmentation would help predict the products that they are more likely to adopt.

**Fig 2:**
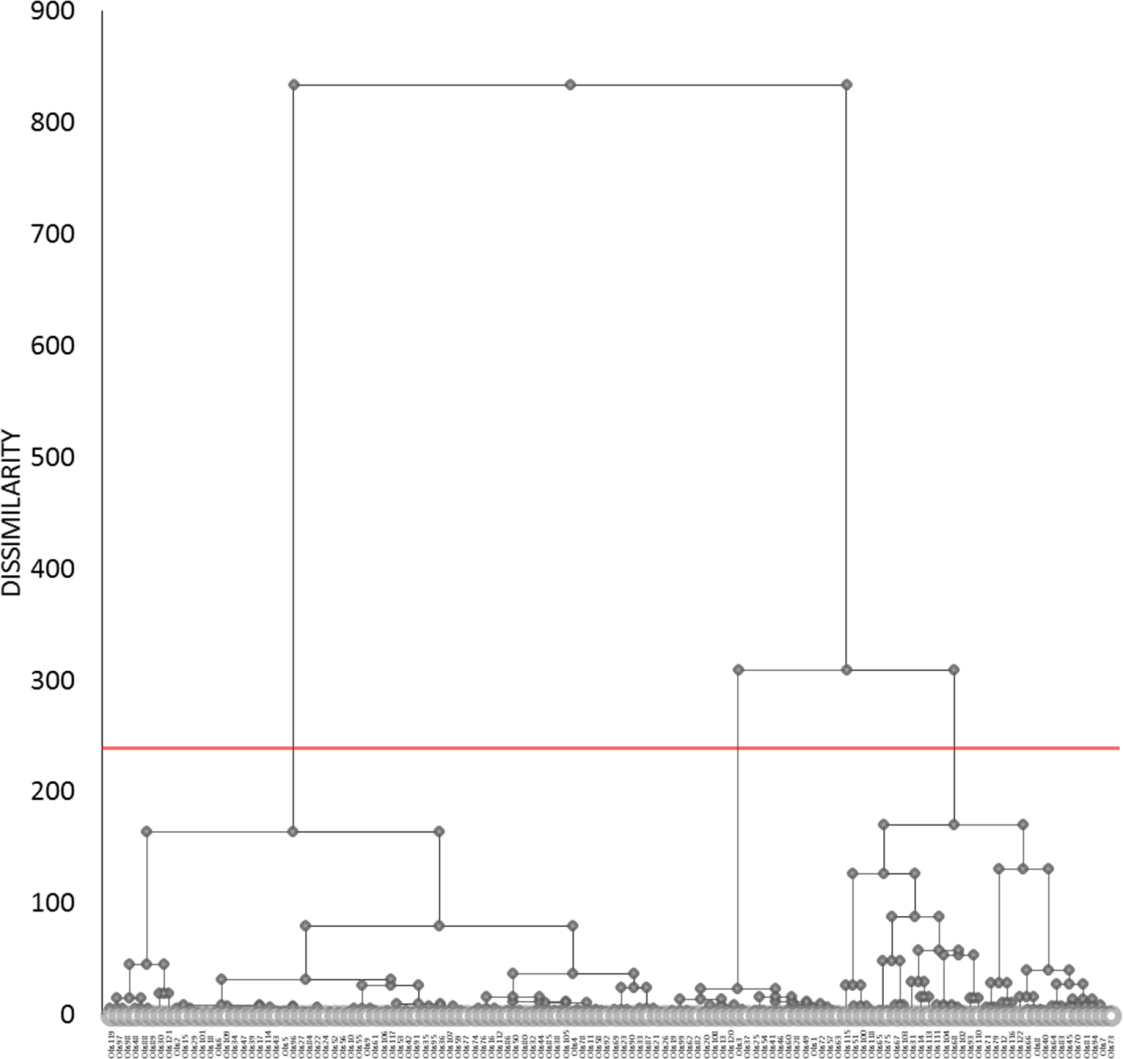
Agglomerative hierarchical cluster analysis dendrogram for segmenting consumers (n = 122) into groups of similar perceptions for fufu and eba products (n = 7). Where: Red line donates level of dissimilarity along which the three segments (groups) were selected.

Cluster analysis indicated three different groups of consumer profile with respect to the fufu and eba products (Fig 2).

**Fig 3:**
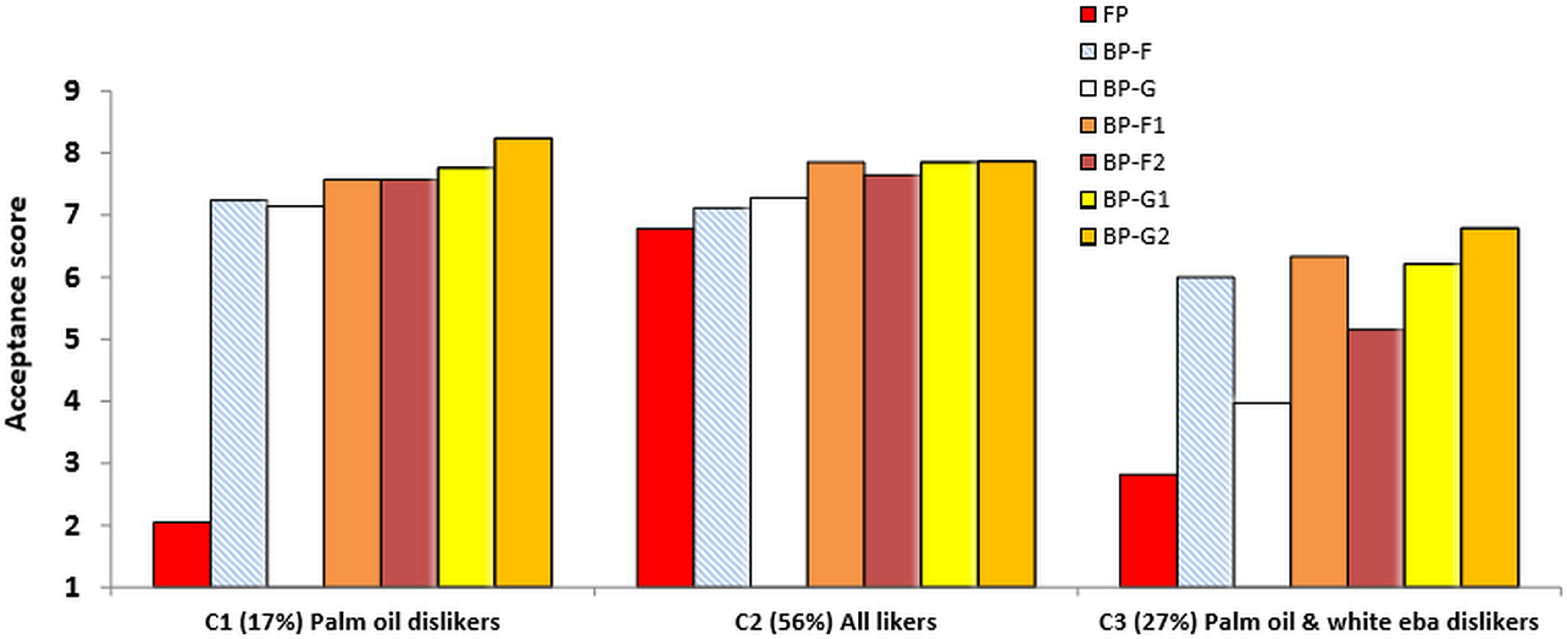
**Mean consumer acceptance score for fufu and eba products by cluster type** (C1; C2 and C3).

The mean liking for each of the three clusters is illustrated in Fig 3.

We used a score of five ‘neither like nor dislike’ as an indicator of “neutral attitude”. The products rated below five were considered as “disliked” and above five as “liked”. For the purposes of cluster division, the groups were grouped as ‘palm oil dislikers’ (Cluster 1 (C1) = 17%), ‘all likers’ (Cluster 2 (C2) = 56%) and ‘palm oil & white eba dislikers’ (Cluster 3 (C3) = 27%). Cluster 2 called ‘all likers’ represented the majority of consumers (56%) and were those who accepted all the types of products (fufu or eba) indifferently. Their hedonic score was high (around 7 and above). Cluster 1 and 3 represented a minority of consumers who had specific liking tastes: Cluster 1 or called ‘palm oil dislikers’ (17% of consumers) found all products very acceptable (score above 7) apart from the product containing red palm oil (RPO) (score ~ 2). Cluster 3 or called ‘palm oil and white eba dislikers’ (27%) were the more selective consumers since they disliked not only the eba with RPO but also the eba from the local (white) variety. Globally their acceptance of the products was lower than C1 and C2 consumers.

Cluster analysis (Wards method) was used to understand the position of different stakeholders from the public and private sector toward genetically modified provitamin A cassava in Brazil [37]. In the study, three groups emerged from the cluster segmentation: Cluster 1 had a positive attitude toward GM food; Cluster 2 a moderate, pragmatic position and Cluster 3, a negative position toward GM crops but no tasting of cultivars was involved. There was a clear segmentation between different groups having different perceptions of GM foods. Cluster analysis was also used to understand market segmentation for high iron biofortified beans in Rwanda [38], and it was shown there were differences in acceptance of the beans in the clusters based on location, consumer’s source of income and nutrition information. Another study [25] used cluster analysis with conventionally bred orange fleshed sweet potato and local sweet potato varieties that were tested by mothers and preschool children and revealed there was no segregation due to the biofortified or non-biofortified nature of the sweet potato.

Our research shows a similar outcome than the latter study in terms of segmentation: the segmentation into clusters shows that white and red palm oil dislikes had an effect on cluster division but there was no liking hindrance with biofortified cassava. This is good news because it shows that biofortified cassava were highly acceptable to adult consumers independently of the cluster they were in.

For other crops such as maize the outcomes can be different: in particular a study on maize [39] in Kenya showed that the colour of biofortified maize (yellow) could be a hindrance to acceptance and willingness-to-pay as opposed to white maize that was fortified or not.

### Relationships between sensory characteristics and consumer acceptability

In order to understand how sensory characteristics influenced on liking, attempts were made to look at correlations using graphic representations as in other publications [29] but there were not clear relationships between sensory attributes scoring and acceptability of varieties. The reason for this could be that most hedonic scores were either high or low (RPO sample) and therefore there was not a good dispersion of data points on correlation graph. Hence other techniques called JAR and CATA were used to link sensory profile of products and consumer acceptability. In our knowledge those techniques have not yet been reported with biofortified foods.

#### Just about right (JAR) analysis

**Fig 4:**
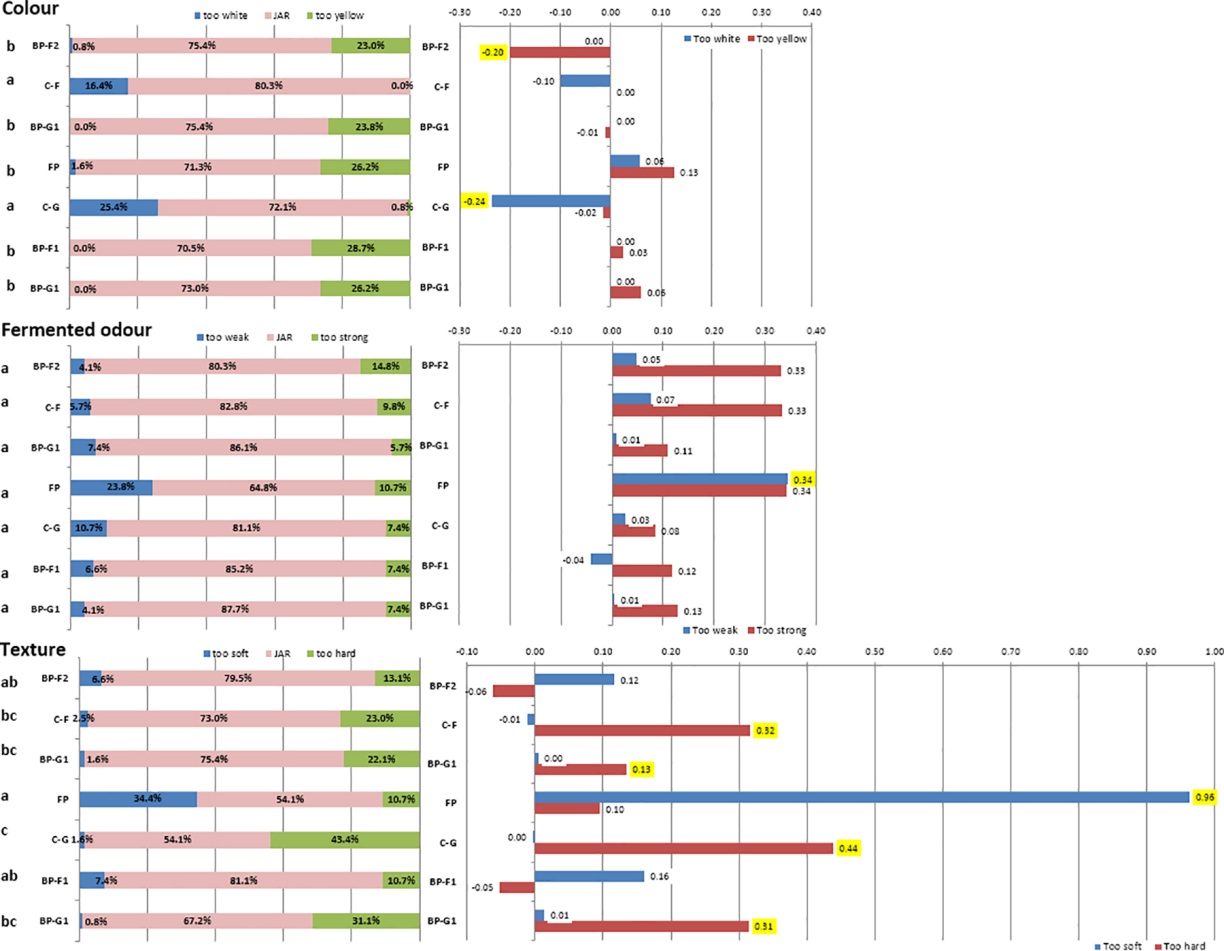
**Frequency scores for Just-about-right (JAR) attributes** (left hand side)**and Mean weight drop** (right hand side) **for the fufu and eba products.** Different letters indicate significant differences between samples at p<0.0001 (Friedman test). Significant Penalty values at p<0.05 are highlighted in yellow (for a minimum of 20% of the consumers). Abbreviations: FP: Eba TME 419 (palm oil); C-G: Eba TME 419; C-F: Fufu TME 419; BP-F2: Fufu 01/1371; BP-G1: Eba 01/1368; BP-F1: Fufu 01/1368; BP-G2: Eba 01/1371.

Percentage answers for colour (too white; JAR; too yellow), fermented odour (too weak; JAR; too strong) and texture (too soft; JAR; too hard) are represented in Fig 4 by product.

Most consumers were satisfied with the sensory characteristics of products they were presented (in pink colour): on average 74.0%, 81.1% and 69.2% of consumers were satisfied with the colour, smell, and odour, respectively.

White eba (C-G) was perceived as ‘too white’, by 25.4% of consumers and biofortified fufu from 01/1371 variety (BP-F2) was considered ‘too yellow’ by 23.0% of consumers but those overstatements actually resulted in improved liking (negative penalty) for those products. This could mean that some consumers liked bright homogeneous colour (independently of the fact it was white or yellow). This observation agreed with studies [40, 41] who reported that fufu of good quality had homogeneous colour: according to Falade & Akingbala [41], good quality fufu could be of various colour: creamy-white, grey or yellow.

There was no overall difference between the samples at p<0.0001 (Friedman test) with regards to fermented odour. The only sample that had significant penalty was FP. The main reasons for the dislike of FP were that odour was perceived as ‘too weak’ by 23.8% of consumers. A minority of consumers (10.7%) considered FP s odour ‘too strong’ but this was not significant according to the Penalty test because the percentage of consumers was less than 20%. This contradicting perception however shows that in any case the odour of FP was not perceived as appropriate and this might have been because of the RPO smell in the sample. In contrast, the white eba from the same variety that did not contain RPO (C-G) was considered satisfying.

Products that were perceived significantly ‘too hard’ were biofortified eba (BP-G1 and BPG2), white fufu (C-F) and white eba (C-G) (31.1%, 22.1%, 23.0% and 43.4% of consumers, respectively). The white fufu (C-F) and eba (C-G) products might have been considered ‘too hard’ because their initial starch content was higher to that of the biofortified varieties, hence their texture was harder after the same length of fermentation [42]. Smoothness is considered an important quality criteria for cassava pastes [42, 43] and most the biofortified products were satisfying to the consumers in terms of their softness or smoothness. On the other hand, texture of FP was perceived as ‘too soft’ (34.4% of consumers) and was strongly penalised. This could be explained by the addition of RPO that softened the texture of FP.

Whilst most of the sensory properties of the other cassava products were considered satisfying, smell and texture of FP were both penalised by the consumers.

#### Check-all-that-apply (CATA) analysis

**Fig 5:**
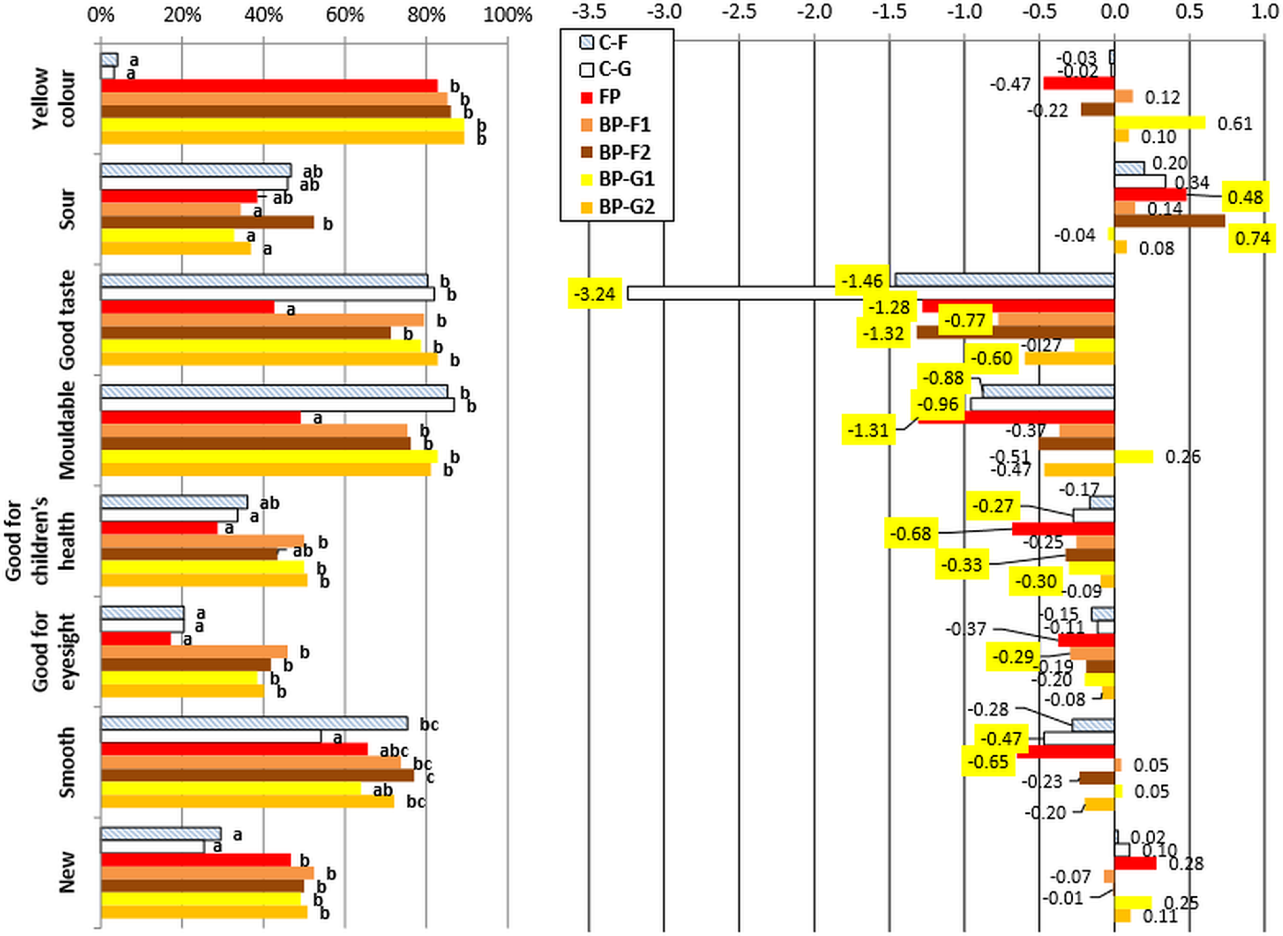
**Frequency scores for Check-all-that-applies (CATA) attributes** (left hand side) **and Mean weight drop** (right hand side). Different letters (a, b, c) indicate significant differences in frequency at p<0.0005 (Cochran’s Q test). Significant Penalty values at p<0.05 are highlighted in yellow. Abbreviations: FP: Eba TME 419 (palm oil); C-G: Eba TME 419; C-F: Fufu TME 419; BP-F2: Fufu 01/1371; BP-G1: Eba 01/1368; BP-F1: Fufu 01/1368; BP-G2: Eba 01/1371.

An analysis of the significance in CATA frequencies using Cochran’s Q test was applied (Fig 5).

All CATA attributes significantly differ (at p<0.05) with respect to the different eba and fufu products. This shows that CATA data could discriminate between different sensory attributes and therefore implies that consumer sensory methods such as CATA could be a viable alternative to sensory panels in sub-Saharan Africa in the same way that it has now been demonstrated in higher income countries [44] on various products [45] including gluten free products [46].

A Penalty analysis investigated the influence of the different CATA attributes selected on consumer liking.

‘Yellow colour’ was clearly identified in yellow eba and fufu products including eba with RPO (FP) as shown by the CATA frequency and this was in accordance with the sensory panel description. ‘Yellow colour’ had no impact on consumer liking. This is good news since it shows that the different colour (yellow) was no hindrance to liking. In addition, there was no effect of the perception of ‘new’ on consumer liking. Hence the colour or novelty perception products did not have an impact on consumer liking.

Frequencies shows that consumers believed that white products were less ‘new’ than yellow products (Cochran’s Q test). It was observed that although FP is a traditional Nigerian product, it was included in the ‘new’ product category: explanations for this may be that FP is less frequently consumed than the white eba in the south-western parts of Nigeria but also yellow colour of the products may have had a bias effect on the perception of novelty by consumers since biofortified products that are new are also yellow.

‘Mouldable’, ‘good taste’, ‘smooth’, ‘good for children ‘health’, and ‘good for eye sight’ attributes all had a positive effect on consumer liking whilst ‘sour’ had a negative impact: two products BP-F2 and FP were penalised for their sourness (Fig 5). If the white cassava products (C-F and C-G) were found ‘mouldable’ this also had a significant positive impact on their liking (‘positive’ penalty). If FP was perceived as ‘mouldable’, ‘smooth’, ‘good for children health’ this gave a significant boost to its acceptance. BP-F1 was the only product that had a positive penalty for ‘good for eyesight’. Perhaps the link between provitamin A content and eye sight was not clear in people’s mind. A major observation was that the frequency of people selecting FP as ‘good for children’s health’ and ‘good for eyesight’ was significantly less than for the BP products (Cochran’s Q test) and was similar to control products (C-G and C-F). In fact FP contains the same level of carotenoids as BP products [9]. It seems therefore that there was further need for education because not all ‘yellow products’ were necessarily clearly associated with nutrition and health in consumer’s mind.

Little research has been reported on the use of CATA on starchy products such as cassava. A research from Benin on traditional gari from white cassava, fortified gari with RPO, and soybean was reported in an international conference on roots and tubers [47]. The study used similar methodologies (hedonic scale, CATA and JAR) with a similar number of consumers interviewed (122). Some of the CATA descriptors were ‘nourishing’, ‘good for health’, ‘attracting’, and ‘good taste’. Those were also close to the descriptors we used although there was no focus on children and eye health. It was concluded that the two fortified gari products did not significantly differ from traditional white gari in terms of acceptance and therefore confirms that fortification with RPO and soyabean was overall well accepted by the population. Unlike this work, our study showed that the RPO-fortified product was less acceptable to the white traditional product. In another study, acceptance of gluten free pasta made from maize from either celiac or non-celiac consumers was explored [46]. The attributes ‘too white’ and ‘smooth’ were used as well as ‘nutritious’, ‘healthy’ and ‘original’ In this study, the ‘nutritious’ and ‘healthy’ attributes were subjective but in our study, the attributes ‘good for children’s health’ and ‘good for eye sight’ relate to actual knowledge of the nutritious benefit of biofortified and RPO products. Results from CATA frequencies show that in spite of nutritional messages delivered to farmers and distribution of biofortified cassava stems, the impact of ‘health’ related attributes on liking and the link with the yellow colour of the cassava product was not always clear to consumers.

## Conclusions

Our study shows that novel biofortified cassava products, namely eba and fufu, were well accepted by local consumers and that yellow colour did not make a difference to acceptability. An interesting observation was that the traditional yellow-coloured RPO-fortified eba product was not as acceptable as the biofortified product of the same colour and this was because the fortified product had less desirable sensory characteristics (possibly due to the addition of palm oil). Good acceptance of biofortified cassava products shows that the barrier to acceptance of novel nutritious food is lower than expected in rural areas of Africa, providing that these products have acceptable sensory characteristics. According to the results of this study, it is anticipated that eba and fufu made from yellow biofortified cassava are likely to be well accepted by local Nigerian populations.

We used a range of methods to describe the sensory and nutritional perception of the products: a classic sensory analysis with a semi-trained panel and two more recent sensory methods with consumers (CATA and JAR). The methods gave consistent results in terms of sensory description of the products. Moreover, the CATA method added another dimension (nutritional perception) to describe products. To our knowledge, it was the first time that the CATA and JAR methods have been used on biofortified crops in a low-and-middle-income- country setting and this shows that there is potential to develop quality nutritional and sensory profiling using consumers with a limited level of formal education.

There was not a clear perception of the link between health benefits (‘good for eye sight’ and ‘good for children’s health’) and the yellow colour of the products. Consumers mistakenly considered that the yellow fortified product was equivalent to white cassava products in terms of health benefits. Hence consumers were not aware of the correlation between yellow colour (in RPO-fortified or biofortified cassava products) and provitamin A. These findings highlight a gap in knowledge in local populations who are beneficiaries of biofortification programs and we advocate for dietary diversification and training programmes that will equally promote biofortified foods and vitamin A-rich- local foods.

## Supporting information

S1 Supporting Information Consumers’ questionnaire. (DOC)

S2 Supporting Information Raw data used for this publication. (XLSX)
