# Supplementary material for: ‘Yellow is good for you’: Consumer perception and acceptability of fortified and biofortified cassava products
Source: PLoS One. 2018 Sep 14;13(9):e0203421. doi: 10.1371/journal.pone.0203421 (PMC6138417; doi:10.1371/journal.pone.0203421)
Supplement: S1 Supporting Information — (DOC) [file pone.0203421.s001.doc]

Date: ______/_____/2013

Place______________ Enumerator____________________

General information

| Age: ______ | Gender: |  Man |  Woman |
| --- | --- | --- | --- |

First Language:

|  Yoruba |  Ibo |  Hausa |  Other. Please indicate______ |
| --- | --- | --- | --- |

Level of education:

|  No education |  Primary |  Secondary |  University |
| --- | --- | --- | --- |

Occupation: ______

**Hedonic tasting (one answer only)**

1^st^ Sample nº __________

 Like extremely

 Like very much

 Like moderately

 Like slightly

 Neither like nor dislike

 Dislike slightly

 Dislike moderately

 Dislike very much

 Dislike extremely

Why? _________

How does this sample compare to your normal eba or fufu?

| **Colour:** |  Too white |  Just perfect |  Too yellow |
| --- | --- | --- | --- |
| **Fermented odour:** |  Too weak |  Just perfect |  Too strong |
| **Texture:** |  Too soft |  Just perfect |  Too hard |

Which of the following applies to the sample, according to your first impression?

|  Yellow colour |  Good taste |  Good for children’s health |  Smooth |
| --- | --- | --- | --- |
|  Sour |  Mouldable |  Good for eyesight |  New |
|  |  |  |  |

**Hedonic tasting (one answer only)**

2^nd^ Sample nº __________

 Like extremely

 Like very much

 Like moderately

 Like slightly

 Neither like nor dislike

 Dislike slightly

 Dislike moderately

 Dislike very much

 Dislike extremely

Why? _________

How does this sample compare to your normal eba or fufu?

| **Colour:** |  Too white |  Just perfect |  Too yellow |
| --- | --- | --- | --- |
| **Fermented odour:** |  Too weak |  Just perfect |  Too strong |
| **Texture:** |  Too soft |  Just perfect |  Too hard |

Which of the following applies to the sample, according to your first impression?

|  Yellow colour |  Good taste |  Good for children’s health |  Smooth |
| --- | --- | --- | --- |
|  Sour |  Mouldable |  Good for eyesight |  New |
|  |  |  |  |

**Hedonic tasting (one answer only)**

3^rd^ Sample nº __________

 Like extremely

 Like very much

 Like moderately

 Like slightly

 Neither like nor dislike

 Dislike slightly

 Dislike moderately

 Dislike very much

 Dislike extremely

Why? _________

How does this sample compare to your normal eba or fufu?

| **Colour:** |  Too white |  Just perfect |  Too yellow |
| --- | --- | --- | --- |
| **Fermented odour:** |  Too weak |  Just perfect |  Too strong |
| **Texture:** |  Too soft |  Just perfect |  Too hard |

Which of the following applies to the sample, according to your first impression?

|  Yellow colour |  Good taste |  Good for children’s health |  Smooth |
| --- | --- | --- | --- |
|  Sour |  Mouldable |  Good for eyesight |  New |

**Hedonic tasting (one answer only)**

4^th^ Sample nº __________

 Like extremely

 Like very much

 Like moderately

 Like slightly

 Neither like nor dislike

 Dislike slightly

 Dislike moderately

 Dislike very much

 Dislike extremely

Why? _________

How does this sample compare to your normal eba or fufu?

| **Colour:** |  Too white |  Just perfect |  Too yellow |
| --- | --- | --- | --- |
| **Fermented odour:** |  Too weak |  Just perfect |  Too strong |
| **Texture:** |  Too soft |  Just perfect |  Too hard |

Which of the following applies to the sample, according to your first impression?

|  Yellow colour |  Good taste |  Good for children’s health |  Smooth |
| --- | --- | --- | --- |
|  Sour |  Mouldable |  Good for eyesight |  New |
|  |  |  |  |

**Hedonic tasting (one answer only)**

5^th^ Sample nº __________

 Like extremely

 Like very much

 Like moderately

 Like slightly

 Neither like nor dislike

 Dislike slightly

 Dislike moderately

 Dislike very much

 Dislike extremely

Why? _________

How does this sample compare to your normal eba or fufu?

| **Colour:** |  Too white |  Just perfect |  Too yellow |
| --- | --- | --- | --- |
| **Fermented odour:** |  Too weak |  Just perfect |  Too strong |
| **Texture:** |  Too soft |  Just perfect |  Too hard |

Which of the following applies to the sample, according to your first impression?

|  Yellow colour |  Good taste |  Good for children’s health |  Smooth |
| --- | --- | --- | --- |
|  Sour |  Mouldable |  Good for eyesight |  New |

**Hedonic tasting (one answer only)**

6^th^ Sample nº __________

 Like extremely

 Like very much

 Like moderately

 Like slightly

 Neither like nor dislike

 Dislike slightly

 Dislike moderately

 Dislike very much

 Dislike extremely

Why? _________

How does this sample compare to your normal eba or fufu?

| **Colour:** |  Too white |  Just perfect |  Too yellow |
| --- | --- | --- | --- |
| **Fermented odour:** |  Too weak |  Just perfect |  Too strong |
| **Texture:** |  Too soft |  Just perfect |  Too hard |

Which of the following applies to the sample, according to your first impression?

|  Yellow colour |  Good taste |  Good for children’s health |  Smooth |
| --- | --- | --- | --- |
|  Sour |  Mouldable |  Good for eyesight |  New |
|  |  |  |  |

**Hedonic tasting (one answer only)**

7^th^ Sample nº __________

 Like extremely

 Like very much

 Like moderately

 Like slightly

 Neither like nor dislike

 Dislike slightly

 Dislike moderately

 Dislike very much

 Dislike extremely

Why? _________

How does this sample compare to your normal eba or fufu?

| **Colour:** |  Too white |  Just perfect |  Too yellow |
| --- | --- | --- | --- |
| **Fermented odour:** |  Too weak |  Just perfect |  Too strong |
| **Texture:** |  Too soft |  Just perfect |  Too hard |

Which of the following applies to the sample, according to your first impression?

|  Yellow colour |  Good taste |  Good for children’s health |  Smooth |
| --- | --- | --- | --- |
|  Sour |  Mouldable |  Good for eyesight |  New |

Could you please rank all the products you have tasted in order of preference (from the most liked (1) to the least liked (7))?

|  | Sample nº |
| --- | --- |
| 1. (most liked) | __________ |
| 2. | __________ |
| 3. | __________ |
| 4. | __________ |
| 5. | __________ |
| 6. | __________ |
| 7. (least liked) | __________ |

**Consumption of cassava-based products**

What is your main staple crop? (tick one answer only)

|  Cassava: |  Maize |  Yam |  Plantain |  Cocoyam |
| --- | --- | --- | --- | --- |
|  Wheat |  Potato |  Rice |  Sweet potato |  |

How do you consume cassava? (tick one answer only)

|  Gari |  Lafun |  Fufu |  Other. Please explain.__________ |
| --- | --- | --- | --- |

**Gari**

Frequency of consumption? (one answer only):

| Times per day? _________ | Times per week? _________ | Times per month? _________ |
| --- | --- | --- |

If you were given a congo of gari, what proportion would you use for the following products?

| Percentage |  |
| --- | --- |
| __________ % | Eba |
| __________ % | Gari with milk/water and sugar |
| __________ % | Gari (dry) |
| 100 % | Total |

What is the colour of the gari that you usually consume?

|  White |  Yellow |  Both |
| --- | --- | --- |

What is your favourite soup for consuming Eba? __________

**Fufu**

Frequency of consumption? (one answer only):

| Times per day? _________ | Times per week? _________ | Times per month? _________ |
| --- | --- | --- |

What is the colour of the fufu that you usually consume?

|  White |  Other. Please explain _________ |
| --- | --- |

What is your favourite soup for consuming Fufu? _________

**Vitamin A cassava**

| Are you aware of vitamin A cassava? |  Yes |  No |
| --- | --- | --- |

If yes, how did you hear about it? (tick one box = most important only)

|  Extension agent |  Radio |  TV |  Newspaper |  friend or neighbour |
| --- | --- | --- | --- | --- |

 Other. Please explain_________

What do you do with vitamin A cassava? (tick one box = most important only)

|  Nothing |  Growing |  Processing |  Eating |  Selling |
| --- | --- | --- | --- | --- |

 Other. Please explain_________

Comments:

………………………………………………………………………………………………………………………………………………
